# A novel case of autogamy and cleistogamy in *Dendrobium wangliangii*: A rare orchid distributed in the dry‐hot valley

**DOI:** 10.1002/ece3.5772

**Published:** 2019-11-06

**Authors:** Qiuxia Wang, Shicheng Shao, Yuan Su, Xueli Hu, Yong Shen, Dake Zhao

**Affiliations:** ^1^ College of Agriculture and Life Sciences Kunming University Kunming China; ^2^ Yunnan Urban Agricultural Engineering & Technological Research Center Kunming China; ^3^ Center for Integrative Conservation Xishuangbanna Tropical Botanical Garden Chinese Academy of Sciences Mengla China; ^4^ Industrial Crop Research Institute Yunnan Academy of Agricultural Science Kunming China; ^5^ College of Agriculture and Biotechnology Yunnan Agricultural University Kunming China; ^6^ Biocontrol Engineering Research Center of Plant Disease & Pest Yunnan University Kunming China; ^7^ Biocontrol Engineering Research Center of Crop Disease & Pest Yunnan University Kunming China; ^8^ School of Life Science Yunnan University Kunming China

**Keywords:** autogamy, cleistogamy, *Dendrobium wangliangii*, drought adaption, fruit set, mating system

## Abstract

*Dendrobium wangliangii* is an epiphytic orchid distributed in the Jinshajiang dry‐hot valley in Luquan County, Yunnan Province, China. Most *Dendrobium* spp. typically have a low fruit set, but this orchid shows a higher fruit set under natural conditions despite the lack of effective pollinators. The pollination biology of the critically endangered *D. wangliangii* was investigated in this study. A fruit set rate of 33.33 ± 4.71% was observed after bagging treatment in 2017 and a high fruit set rate (65.72 ± 4.44% in 2011; 50.79 ± 5.44% in 2017) was observed under natural conditions, indicating that *D. wangliangii* is characterized by spontaneous self‐pollination. The anther cap blocked the growing pollinium; thus, the pollinium slid down and reached the stigmatic cavity, leading to autogamous self‐pollination. Specifically, 51.50% of 162 unopened flowers (total 257 flowers) of this *Dendrobium* species under extreme water‐deficit conditions developed into fruits, suggesting the presence of cleistogamy in *D. wangliangii*. Here, cleistogamy may represent the primary mode of pollination for this orchid. Spontaneous self‐pollination and specific cleistogamous autogamy could represent major adaptions to the drought and pollinator‐scarce habitat in the Jinshajiang dry‐hot valley.

## Graphical abstract

*Dendrobium wangliangii* is an epiphytic orchid distributed in the Jinshajiang dry‐hot valley in Luquan County, Yunnan Province, China. *D. wangliangii* is characterized by spontaneous self‐pollination and cleistogamy. This is a novel case on cleistogamous autogamy in *Dendrobium*.

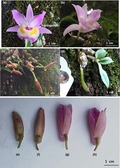

## 1 INTRODUCTION

The floral diversity and pollination biology of Orchidaceae have long intrigued evolutionary biologists (Cozzolino & Widmer, 2005). Orchids are well known for their highly evolved flowers that promote cross‐pollination. Most orchid species rely on pollinators for sexual reproduction (Jacquemyn, Micheneau, Roberts, & Psiller, 2005; Ren, Wang, & Luo, 2012). However, not all orchids possess adaptations that guarantee cross‐pollination, and some have become modified to ensure self‐pollination. Autogamy is the preferred mode of pollination and is a rather common phenomenon within the orchid family (Kowalkowska & Margońska, 2012). Between 5% and 20% of orchid species have evolved autogamy (Gale, 2007; Pang et al., 2012; Tremblay, Ackerman, Zimmerman, & Calvo, 2005). This mode of autogamy often occurs under windless conditions and in drought and insect‐scarce habitats to ensure reproductive success (Liu et al., 2006).

Cleistogamy is a type of special autogamy. It refers to a pollination system in which unopened flowers are capable of autogamy resulting in fruit productions. This trait is an adaptive mechanism for retaining reproduction under poor growth conditions, such as drought, extreme airflow, and sunlight (Faisal et al., 2018; Liu et al., 2006; Lord, 1981; Zou, Zhou, Angessa, Zhang, & Li, 2018). In a majority of cleistogamous species, closed (cleistogamous, CL) and open flowers (chasmogamous, CH) usually occur on the same individuals during a single flowering season. These flowers comprise multiple strategies and perform the same function simultaneously (Schoen & Lloyd, 1984). Both CL and CH flowers produce fruit and seed depending on environmental conditions, including parameters such as light intensity, photoperiod, water availability and nutrient availability (Morinaga et al., 2008).

Generally, *Dendrobium* spp. flower much often but exhibit a low fruit set rate; the fruit set rate of natural populations of *Dendrobium* is approximately 10% to 17% (Zhang, Xia, Zhu, & Zhang, 2000). The low rate of fruit set is mainly attributed to insect‐scarce habitats. Furthermore, self‐incompatibility is found in approximately 50% of *Dendrobium* species. Most *Dendrobium* species undergo cross‐pollination (Brodmann et al., 2009; Niu et al., 2017). The restriction of low numbers of pollinating insects is the main factor leading to low fruit set rates in the field. *Dendrobium wangliangii*, a recently described species of *Dendrobium* (Hu, Long, & Jin, 2008), endemic to the Jinshajiang dry‐hot valley in Luquan County, Yunnan Province, and found at an altitude of 2,200 m, is an epiphytic orchid with strong drought tolerance (Zhao et al., 2013, 2018). Studies on this plant in the wild observed that the rate of fruit set of *D. wangliangii* was rather high under natural conditions.

A common trend of pollinator scarcity is noted under drought conditions (Liu et al., 2006), that may exert selective pressure via pollinator limitations, thus promoting the evolution of autogamy as a reproductive assurance mechanism. Therefore, the reproductive biology of *D. wangliangii* represents an interesting problem. In the present study, *D. wangliangii* was used to investigate the following questions based on preliminary observations: (a) What type of mating system does *D. wangliangii* possess? and (b) If the species exhibits self‐pollination, what type of pollination strategy is employed?

## 2 MATERIALS AND METHODS

### 2.1 Study sites and species

Studies were conducted in one wild population in northern Yunnan province, China. The exact location of *D. wangliangii* is not provided here due to the concern of illegal collection of this endangered orchid. The study area has a subtropical monsoon climate with a mean 947.7 mm of rainfall and average temperature of 15.6°C per year. The forest vegetation at this location is mainly composed of *Quercus* species and *D. wangliangii* epiphytes on these deciduous *Quercus*.

To compare the mating system of *D. wangliangii* with other species, 23 *Dendrobium* spp. (Table 3) were selected and collected from Yunnan, Southeast Asia, and other surrounding regions. These species are cultivated in the nursery garden in Kunming, Yunnan, China. The study area has a monsoonal climate in the northern subtropical low‐latitude plateau with a mean 1,031 mm of rainfall and an average temperature of 13.2°C per year.

### 2.2 Floral morphology and phenology

For investigation of the floral morphology, 30 fresh flowers on 20 different unopened inflorescences (20 plants) were selected at random. The color, morphology, number of stem nodes, and number of flowers on the stem node were recorded and photographed. Flower scent was determined by sniffing with nose. Nectar was observed with naked eyes and then collected with glass capillaries at different flowering stages. The following parameters were measured with vernier calipers (0.01 mm): the length and width of a petal, the diameter of a labellum, the distance between two patches (greenish yellow variegations on either side described by Hu et al. (2008), the length of a gynandrium, and the vertical distance between the stigma and labellum.

Flowering phenology of *D.  wangliangii* was observed in May 2011 and 2017, and the beginning, peak, and end time of the flowering period were recorded. Sixty unopened flowers on 30 inflorescences in the bud stage (30 plants) were randomly marked and subsequently observed. The opening and wilting time of each flower, the number of blooming flowers, and unopened flowers under natural conditions were documented every 2 hr from 6:00 to 18:00 before flower withering. The standard definition of a blooming flower is the spread of the middle labellum, which allows pollinators to visit flowers, and the standard definition of a withering flower is the change of perianth color and morphology.

### 2.3 Flower visitors and their visiting behavior

To record flower visitors and their visiting behavior, the study site was observed during the flowering periods of *D. wangliangii* for a total of 120 hr. Thirty blooming flowers were randomly marked and subsequently observed. Daily periods of observation were from 6:00 to 18:00. Visiting behavior of insects was recorded and photographed, and the following factors were noted: visitor species, visitor time, and visitor behavior, especially if the visitors take pollinia away.

### 2.4 Mating system test

A series of experiments were conducted in 2011 and 2017 to investigate the mating system of *D. wangliangii*. In each experiment, 10 flowers were chosen at random on the first day of anthesis in three replicates (total 30 flowers). Hand pollinations and emasculations were performed using a dissecting needle, and the pollination groups were as follows: (1) artificial self‐pollination by directly transferring the pollinia onto the stigma of the same flower, (2) hand cross‐pollination using pollinia from donor plants that grow at least 3 m away from the plants, (3) spontaneous self‐pollination (mechanical autogamy) by covering the buds under investigation with a fine mesh polyester bag that was subsequently sealed; the self‐pollination strategy was observed by dissection of the flowers in the stages ranging from bud to withering, (4) agamospermy by bagging experiments after emasculating pollinium, to determine whether is *D. wangliangii* parthenogenesis, thus confirming the presence of autogamy, (5) control treatment by marking flowers without any manipulation. The fruit set of the experimental series and that of untreated flowers was recorded in July.

The study of the mating system of 23 *Dendrobium* spp. was performed at the nursery from May to September 2017. Experimental treatments included hand self‐pollination, and hand cross‐pollination, controls, the same to treatments 1, 2, and 5 as described above for *D. wanglianglii*. Ten flowers on 10 inflorescences from five plants per species were randomly selected each time on the morning on their third day of anthesis, with three replicates. Pollination success was estimated with the fruit set after the following 90 days. The fruit set rate was calculated as a number of capsules per number of treated flowers.

### 2.5 Evaluating fruit set under natural conditions

A total of 257 buds of *D. wangliangii* were randomly selected and marked. Within the next two weeks, the open state of the marked flowers was recorded. If the marked flowers remained closed throughout the flowering period, they were marked as unopened; on the contrary, they were marked as opened. After two months, the fruit set rate of the flowers in different states was calculated (as a number of capsules that produced fruit per number of marked flowers).

### 2.6 Statistical analyses

For mating system assays, experiments were conducted by different treatments. Fruit set rate of each treatment was used for statistical analyses. Comparison between the different treatments was performed via one‐way ANOVA and LSD with SPSS (ver. 19.0).

## 3 RESULTS

### 3.1 Floral morphology and phenology

**Figure 1:**
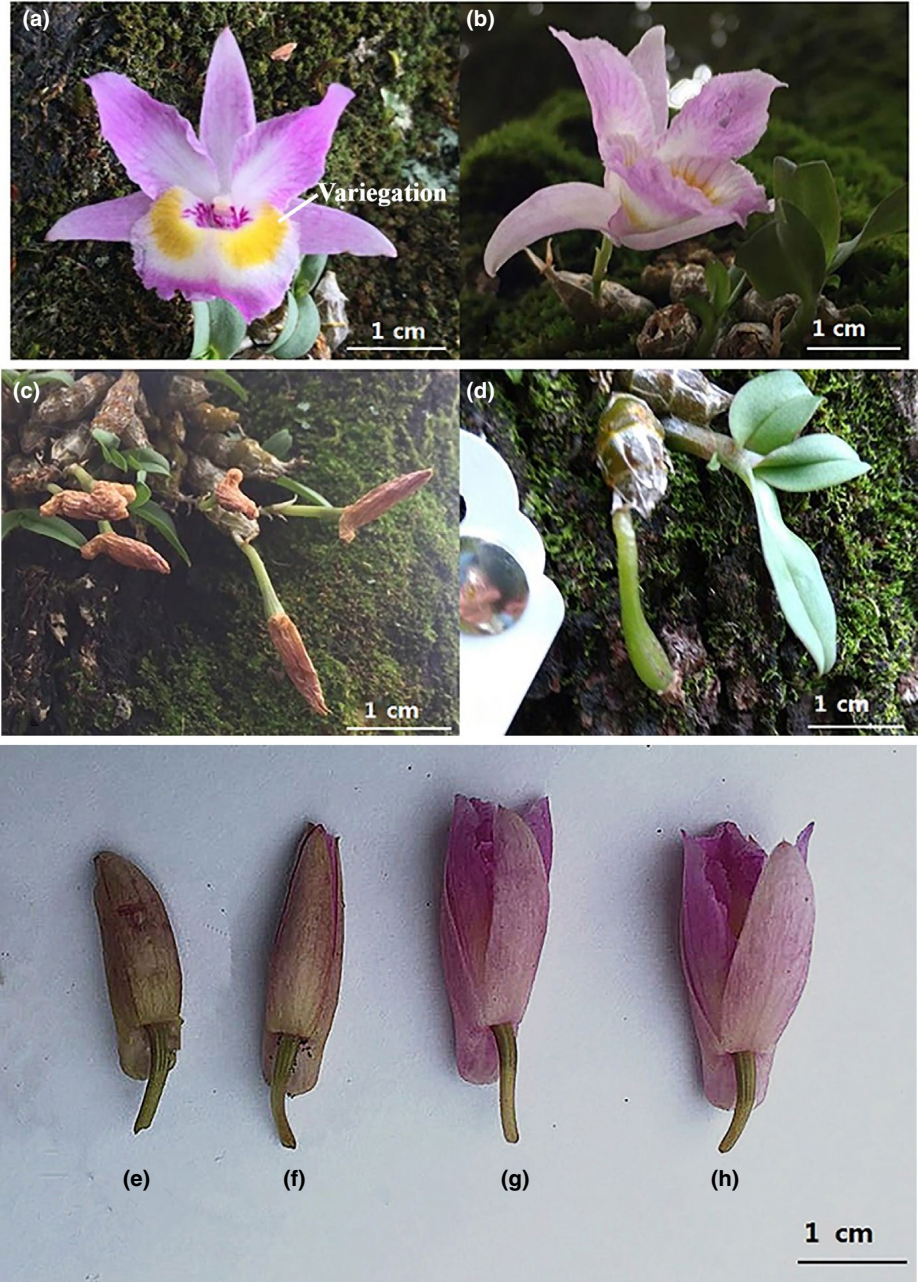
The flowers, fruits, and buds of *Dendrobium wangliangii*. (a) Flower morphological characteristics; (b) flowers growing on the stem nodes; (c) ovary developing and swelling in unopened flowers; (d) fruits of *Dendrobium wangliangii*; (e–h) the buds of *Dendrobium wangliangii* before dissection

*Dendrobium wangliangii* flowers are pink (red‐lilac) with two yellow (greenish yellow) patches on the labellum (Figure 1a). The flower was borne at the upper nodes of an older leafless stem occasionally with two or more flowers on a single inflorescence. The mean number of flowers on a single inflorescence was 1.04 ± 0.05 (*n* = 520). Inflorescences were mostly borne on the first or second stem node (Figure 1b), and a few inflorescences were borne on the third node. According to plant age and number of branches, the number of flowers per plant ranged from 0 to 22. The number of fruits per plant is 2.04 ± 0.12 (*n* = 254). The most common number of fruits observed was 1 to 3 (Figure 1d), and up to 17 fruit sets were noted per plant.

In this study, the length and width of petals were 30.83 ± 0.89 mm and 23.97 ± 0.41 mm, respectively. The diameter of the labellum was 6.99 ± 0.08 mm (Table S1). Other morphological characteristics were detailed in Table S1.

The flowering period of *D. wangliangii* began in early June. The period from the formation to the split of the bud was approximately 2 months. Subsequently, the patches on the labellum gradually appeared with the opening of the bud split; then, flowering reached peak level. Scent was not sniffed and nectar was not observed in the glass capillaries at different flowering stages. However, not all of the flowers could reach the peak, and the full open rate in the population was 42.26 ± 13.34% (*n* = 549). Some buds withered before the appearance of patches which was followed by the ovary beginning to develop and swell (Figure 1c). Most of the flowers facing sunward withered in 1–2 days, and the florescence in the shade lasted for up to 5 days. The average lifespan of a single flower was 2.33 ± 0.15 days (*n* = 62). Under natural conditions, an unfertilized flower could generally remain open for approximately 15 days. After successful pollination, the labellum shrank, and the flower withered.

### 3.2 Flower visitors and their visiting behavior

Two types of visitors were found, that is, fly and the cockchafer. However, the insects did not enter the flower passage of *D. wangliangii* but only crawled outside the flowers, leaving after a few seconds. During the observation period, the pollens removal by any insect and pollination were never observed during either the day or night. In the natural habitat, effective pollinators were scarce, and no insect was observed pollinating *D. wangliangii*.

### 3.3 Mating system

The fruit set rate was 65.72 ± 4.44% under artificial self‐pollination in 2011, which was the highest in all treatments. The difference between different treatments was not significant in 2011 (*p *> .05). In 2017, the difference was not significant between artificial self‐pollination and natural pollination (*p *> .05). There was no difference between hand cross‐pollination and agamospermy (*p *> .05). The difference between other treatments was statistically significant (*p* < .05). The rates of fruit set under hand and spontaneous self‐pollination were significantly higher than that under hand cross‐pollination in 2017 (*p* < .05). Fruits were not formed under agamospermy in *D. wangliangii* (Table 1).

**Table 1:** Mating system of *Dendrobium wangliangii*

Treatment	2011			2017		
	No. of flowers	No. of fruits	Fruits set rate (%)	No. of flowers	No. of fruits	Fruits set rate (%)
Artificial self‐pollination	36	30	83.04 ± 7.25a	45	30	65.36 ± 9.68a
Hand cross‐pollination	30	19	63.07 ± 7.05a	71	6	10.98 ± 1.75c
Spontaneous self‐pollination	—	—	—	30	10	33.33 ± 4.71b
Agamospermy	30	0	0a	30	0	0c
Natural pollination	50	33	65.72 ± 4.44a	61	44	50.79 ± 5.44a

Both opened and unopened flowers of *D. wangliangii* developed into fruits (Table 2). The rate of fruit set displayed no significant difference between opened and unopened flowers (*p *> .05). The unopened flowers produced fruits, indicating that *D. wangliangii* exhibited cleistogamous reproduction.

**Table 2:** Natural fruit set rate of *Dendrobium wangliangii* at different opening stages

State	No. of flowers	No. of fruits	Fruit set rate (%)
Opened	95	51	53.37 ± 9.54a
Unopened	162	76	51.50 ± 7.19a

In nursery experiments, fruit set in 14 *Dendrobium* spp. was formed under both self‐ and cross‐pollination, while fruit sets in nine species did not form under self‐pollination (Table 3). No fruits were formed under the control treatment in 23 *Dendrobium* species. In this experiment, the rates of fruit set under self‐pollination and cross‐pollination were rather high in *Dendrobium nobile* (51.21% and 66.67%, respectively), *Dendrobium trantuanii* (85.71% and 78.57%, respectively), and *Dendrobium trigonopus* (73.33% and 62.24%, respectively). However, the fruit set rates under cross‐pollination were very high in *Dendrobium jenkinsii* (53.33%), *Dendrobium loddigesii* (42.22%), *Dendrobium chrysotoxum* (50.91%), and *Dendrobium primulinum* (47.62%), while the fruit set rates for these species were relatively low under self‐pollination (Table 3).

**Table 3:** The mating systems and fruit set rates for 23 selected *Dendrobium* spp.

Species	Locality	Treatments	No. of flowers	No. of fruits	Fruit set rate (%)
*Dendrobium nobile*	Yunnan, Southeast Asia, Guangxi, Sichuan, Guizhou	Self‐pollination	31	16	51.21 ± 8.70
		Cross‐pollination	30	20	66.67 ± 7.20
*Dendrobium jenkinsii*	Yunnan, Southeast Asia	Self‐pollination	32	6	18.79 ± 4.31
		Cross‐pollination	30	16	53.33 ± 5.44
*Dendrobium loddigesii*	Yunnan, Southeast Asia, Guangxi, Guangdong, Guizhou	Self‐pollination	30	5	16.67 ± 12.47
		Cross‐pollination	32	14	42.22 ± 17.31
*Dendrobium wardianum*	Yunnan, Southeast Asia	Self‐pollination	30	4	13.33 ± 2.72
		Cross‐pollination	33	6	17.73 ± 3.54
*Dendrobium trantuanii*	Vietnam	Self‐pollination	22	19	85.71 ± 6.73
		Cross‐pollination	23	18	78.57 ± 6.57
*Dendrobium denneanum*	Yunnan, Burma, Taiwan	Self‐pollination	32	7	21.52 ± 4.70
		Cross‐pollination	30	8	26.67 ± 2.72
*Dendrobium findlayanum*	Yunnan, Southeast Asia	Self‐pollination	32	8	24.85 ± 1.98
		Cross‐pollination	30	5	16.67 ± 5.44
*Dendrobium chrysotoxum*	Yunnan, Southeast Asia	Self‐pollination	35	2	5.13 ± 4.19
		Cross‐pollination	33	16	50.91 ± 8.91
*Dendrobium capillipes*	Yunnan, Southeast Asia	Self‐pollination	30	18	60.00 ± 4.71
		Cross‐pollination	31	18	44.24 ± 12.55
*Dendrobium cariniferum*	Yunnan, Southeast Asia	Self‐pollination	30	2	6.67 ± 9.34
		Cross‐pollination	30	2	6.67 ± 5.44
*Dendrobium trigonopus*	Yunnan, Southeast Asia	Self‐pollination	30	22	73.33 ± 9.81
		Cross‐pollination	31	20	64.24 ± 5.85
*Dendrobium crepidatum*	Yunnan, Southeast Asia, Guizhou	Self‐pollination	30	10	33.33 ± 7.20
		Cross‐pollination	31	7	22.42 ± 5.11
*Dendrobium primulinum*	Yunnan, Southeast Asia	Self‐pollination	8	2	33.33 ± 7.20
		Cross‐pollination	23	11	47.62 ± 1.94
*Dendrobium tortile*	Yunnan, Southeast Asia	Self‐pollination	32	4	12.42 ± 4.09
		Cross‐pollination	35	9	25.26 ± 2.54
*Dendrobium guangxiense*	Yunnan, Guangxi, Guizhou	Self‐pollination	31	0	0
		Cross‐pollination	31	21	70.61 ± 17.12
*Dendrobium hancockii*	Yunnan, Guangxi, Sichuan, Guizhou	Self‐pollination	33	0	0
		Cross‐pollination	32	14	43.33 ± 5.44
*Dendrobium cucullatum*	Yunnan, Southeast Asia, Guangxi	Self‐pollination	31	0	0
		Cross‐pollination	34	9	28.33 ± 14.21
*Dendrobium hainanensis*	Yunnan, Southeast Asia	Self‐pollination	17	0	0
		Cross‐pollination	18	14	83.33 ± 13.61
*Dendrobium dixanthum*	Yunnan, Southeast Asia	Self‐pollination	19	0	0
		Cross‐pollination	10	0	0
*Dendrobium thyrsiflorum*	Yunnan, Southeast Asia	Self‐pollination	33	0	0
		Cross‐pollination	32	0	0
*Dendrobium ratiosissimum*	Yunnan, Southeast Asia	Self‐pollination	30	0	0
		Cross‐pollination	30	0	0
*Dendrobium amabile*	Yunnan, Hainan, Vietnam	Self‐pollination	45	0	0
		Cross‐pollination	37	0	0
*Dendrobium ochreatum*	Southeast Asia	Self‐pollination	6	0	0
		Cross‐pollination	21	9	43.45 ± 2.96

Nine *Dendrobium* spp. were self‐incompatible, while five orchids, including *Dendrobium ochreatum*, *Dendrobium guangxiense*, *Dendrobium hancockii*, *Dendrobium cucullatum*, and *Dendrobium hainanensis*, were cross‐compatible. The fruit set rates under cross‐pollination were high (Table 3).

The fruit set rate under cross‐pollination was significantly higher than that under self‐pollination in 23 *Dendrobium* spp. (*p* < .05), revealing that the mating system of *Dendrobium* was dominated by cross‐pollination (Table 4).

**Table 4:** Variance analysis of self‐pollination rate and cross‐pollination rate

	Sum of squares	*df*	Mean square	*F*	Sig.
Between groups	3,241.561	1	3,241.561	4.933	0.032
Within groups	28,915.872	44	657.179		
Total	32,157.434	45			

**Figure 2:**
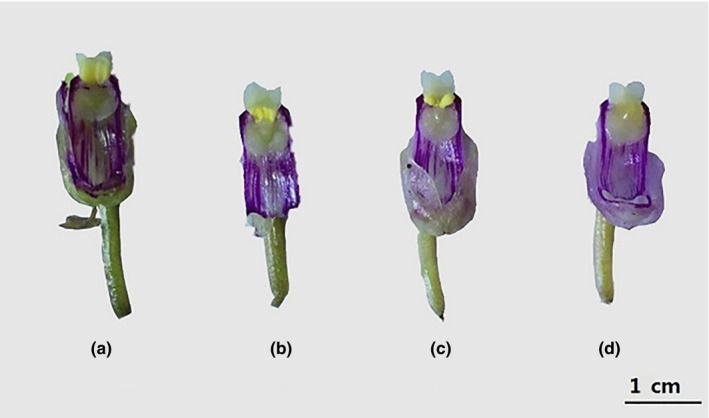
The process of automatic pollination of *Dendrobium wangliangii*. (a) The anther cap was covered with pollen, and the pollen did not adhere to the stigma, exhibiting a pale yellow color; (b) the anther cap blocked the growing pollinium and the pollinium slid down and reached the stigmatic cavity; (c) after the pollen grains absorbed water on the stigma, their volume expanded; (d) the pollen grains were gradually integrated into the stigma and the color faded

Buds' morphology was observed by dissection in *D. wangliangii* (Figure 1e–h). Under natural conditions, the anther cap was covered with pollen, and the pollen did not adhere to the stigma, exhibiting a pale yellow color (Figure 2a). Thereafter, the anther cap blocked the growing pollinium; thus, the pollinium slid down and reached the stigmatic cavity (Figure 2b). After the pollen grains absorbed water on the stigma, their volume expanded (Figure 2c). Subsequently, the pollen grains were gradually integrated into the stigma (Figure 2d).

## 4 DISCUSSION

The present investigation provides evidence for autonomous self‐pollination in *D. wangliangii*. Self‐compatibility is thought to be relatively common in orchids, while only 10% of orchid species exhibit self‐incompatibility (SI). Self‐compatibility has few been reported in a typical genus with SI, *Dendrobium* (Catling, 1990; Liu et al., 2006; Niu et al., 2017).

*Dendrobium wangliangii* was self‐compatible but also cross‐compatible for fruit set, and the fruit set rate under hand self‐pollination was higher than that under hand cross‐pollination. Inversely, the fruit set rate under cross‐pollination was significantly higher than that under self‐pollination in 23 tested *Dendrobium* spp., revealing that *Dendrobium* mating system mainly involved cross‐pollination. The fruit set rate under spontaneous self‐pollination reached to 33.33%, which suggested that *D. wangliangii* displayed the trait of autonomous self‐pollination. A majority (72%) of *Dendrobium* species exhibits SI (Niu et al., 2017), so autonomous self‐pollination could represent a unique mating system in *Dendrobium* spp., including *D. wangliangii*. Niu et al. (2017) found that the orchid S‐RNase‐like genes were not specifically expressed, suggesting that these genes might not be involved in orchid SI. Based on previous phylogenetic analyses, *Dendrobium* was divided into two main clade: Asian clade and Australasian clade (Xiang et al., 2016), and eight *Dendrobium* spp. (including *D. wangliangii*) whose mating systems we have tested were assigned into the Asian clade. *D. wangliangii* was showed to be most relative to *D. devonianum* while far from the other seven evaluated *Dendrobium* species (*D. nobile*, *D. loddigessi*, *D. findlayanum*, *D. chrysotoxum*, *D. tortile*, *D. hancockii*, *D. ochreatum*; Xiang et al., 2016). *D. hancockii* and *D. ochreatum* displayed cross‐compatible and self‐incompatible. Similar to the other five species (*D. nobile*, *D. loddigessi*, *D. findlayanum*, *D. chrysotoxum*, *D. tortile*), *D. devonianum* and *D. wangliangii* were self‐compatibility but also cross‐compatible (Li et al., 2009; Lian & Li, 2003). Furthermore, *D. wangliangii* evolves a trait of autonomous selfing. Therefore, it is tempting to speculate that *D. wangliangii* is a special case of evolution to autogamy. Darwin (1890) realized that autogamy could represent an adaptation to reproduction if pollinator service was lost or extremely unpredictable, thus providing reproductive assurance. Both abiotic and biotic factors can influence mating‐system evolutionary transitions from outcrossing to selfing, such as pollinator absence or low pollinator abundance (Kalisz & Vogler, 2003). Fishman and Willis (2008) reported that pollen limitation intensified selection on attractive floral traits with increasing pollen limitation, but severe pollen limitation may favor traits associated with autonomous self‐fertilization. Here, autogamous self‐pollination of *D. wangliangii* may be an adaptive evolution to the orchid's dry and insect‐limited habitat.

No effective pollinator was observed in our investigation. Most Orchidaceae species exhibit particular types of floral morphology, color patterns, fragrances, or rewards for cross‐pollination; thus, the tendency of automatic self‐pollination is reduced (Kowalkowska & Margońska, 2012). However, automatic self‐pollination within Orchidaceae is a rather common mode in windless, drought conditions, and insect‐limited habitats (Liu et al., 2006). Numerous studies have documented the tendency of plants to autonomous self‐pollination when grown in dry and insect‐limited conditions (Dole, 1990; Elle & Carney, 2003; Elle & Hare, 2002; Herrera et al., 2001; Holsinger, 2000; Liu et al., 2006). Flowers can attract pollinators based upon attraction and reward (Monty, Saad, & Mahy, 2006; Pang et al., 2012). However, in the study, *D. wangliangii* lacked nectar and odors. The loss of attraction and reward for *D. wangliangii* may lead to a deficiency of effective pollinators under natural conditions, which was coincident with previous findings demonstrating a common trend of pollinator scarcity in drought conditions and high mountains (Blionis & Vokou, 2008; Li, Zheng, Dafni, & Luo, 2010; Liu et al., 2006; Totland, 2001). Pollinator scarcity may drive evolutionary shift from cross‐pollination to self‐pollination (Xiong, Fang, & Huang, 2013). Most of the mycoheterotrophic species (especially nectarless species) seem to have abandoned insect pollinators in favor of self‐pollination (Suetsugu, 2016). In *D. wangliangii*, autonomous self‐pollination could represent an evolutionary response to ensure reproductive success to compensate for pollinator limitation.

Cleistogamy is rather common in orchids (Culley & Klooster, 2007), but this study represents the first time cleistogamy is reported in *D. wangliangii*. In our study, the unopened flowers developed into fruits, suggesting that *D. wangliangii* could produce fruit via a cleistogamous trait. Cleistogamy is a special form of automatic self‐pollination that is likely initiated and regulated by different habitat types, such as temperature, photoperiod, or moisture (Berg & Redbo‐Torstensson, 1998; Schemske, 1978). In the study, the average florescence of *D. wangliangii* was 2.33 ± 0.15 days. Compared with other *Dendrobium* species (florescence: 15–30 days; Gong, Wang, Sun, Lin, & Yang, 2013), the florescence of the orchid was very short. In general, cleistogamy is favored under adverse and stressful conditions (e.g., scarce‐pollinator, water deficiencies), which are unsuitable for cross‐pollination (Schemske, 1978; Solbrig, 1976). A short florescence may be adverse for cross‐pollination. Thus, the orchid could evolve a particular mating system, namely cleistogamy. This mode may be beneficial to enhance the adaptability of *D. wangliangii* to adverse environments, such as drought, short florescence, and pollinator‐scarce conditions (Abdel‐Ghani, Parzies, Omary, & Geiger, 2004; Miranda & Vieira, 2014). In addition, cleistogamy reduces the threat of genetic contamination and gene flow through crossing with other plants of the same species (Faisal et al., 2018; Travers, Anderson, Vitt, & Harris, 2018; Zou et al., 2018). Outcrossing has a selective advantage, such as maintaining high genetic diversity and avoiding sibling competition (Antlfinger, 1986; Cozzolino & Widmer, 2005; Culley & Klooster, 2007; Schmitt, Ehrhardt, & Swartz, 1985). Commonly, selfing is considered to decrease genetic variation and increased genetic drift, high levels of inbreeding depression (Culley & Klooster, 2007). However, when pollinators are rare or absent, cleistogamous selfing can exhibit fitness advantages distinct from out‐crossing, owing to the increase of seed production, less energetical cost, and inherent automatic selfing advantage (Culley & Klooster, 2007; Suetsugu, 2014, 2016; Waller, 1984). Outcrossing in the orchid may gradually lose its position under pollinators scarcity and extreme water‐deficit conditions. Finally, *D. wangliangii* may evolve a specific mating system for reproductive assurance, cleistogamy. The breeding system evolved at least six to eight times within monocots (Culley & Klooster, 2007; Hilu et al., 2003; Soltis et al., 2000). Consistent cleistogamous selfing can eliminate deleterious recessive alleles within populations; over time, this could lead to a decrease in the level of inbreeding depression (Clay & Antonovics, 1985; Culley & Klooster, 2007). Here, cleistogamy in *D. wangliangii* could ensure population purity and germplasm stability. The evolutionary pathway to cleistogamy remains relatively understudied, so cleistogamous species can be used to test evolutionary theories and origins of cleistogamous taxa.

*Dendrobium wangliangii* thus exemplifies a unique mode of autogamy, cleistogamy, in the genus *Dendrobium*. This can provide a novel breeding mechanism in *Dendrobium*. Future research will explore the importance of climate and development time for mating system evolution, the extent of dependency on autonomous self‐pollination for reproductive success and the molecular mechanism of cleistogamy in this species.

## CONFLICT OF INTEREST

None declared.

## AUTHOR CONTRIBUTIONS

Shicheng Shao, Yuan Su, and Xueli Hu conducted the field and greenhouse research. Qiuxia Wang performed data analysis and wrote the manuscript. Dake Zhao and Yong Shen designed the research. All authors read and approved the final manuscript. Qiuxia Wang and Shicheng Shao contributed equally to this work.

## Data Availability

The mating systems, fruit set rates, and morphological characteristics data used in the manuscript can be accessed at the Dryad Digital Data Repository: Dryad https://doi.org/10.5061/dryad.11n8t6n.
