# Supplementary material for: A novel case of autogamy and cleistogamy in Dendrobium wangliangii: A rare orchid distributed in the dry‐hot valley
Source: Ecol Evol. 2019 Nov 6;9(22):12906–14. doi: 10.1002/ece3.5772 (PMC6875582; doi:10.1002/ece3.5772)
Supplement: Supplementary file 1 [file ECE3-9-12906-s001.docx]

**Table S1 Morphological characteristics of *Dendrobium wangliangii***

| **Morphological characteristics** | **Number** | **Mean**±**SD (mm)** |
| --- | --- | --- |
| Petal length | 30 | 30.83±0.89 |
| Petal width | 30 | 23.97±0.41 |
| labellum diameter | 30 | 6.99±0.08 |
| Distance between two patches | 30 | 3.49±0.08 |
| Gynandrium length | 30 | 6.18±0.07 |
| Vertical distance between stigma and labellum | 30 | 6.18±0.07 |
